# *Bacillus circulans* GN03 Alters the Microbiota, Promotes Cotton Seedling Growth and Disease Resistance, and Increases the Expression of Phytohormone Synthesis and Disease Resistance-Related Genes

**DOI:** 10.3389/fpls.2021.644597

**Published:** 2021-04-14

**Authors:** Lijun Qin, Peidong Tian, Qunyao Cui, Shuping Hu, Wei Jian, Chengjian Xie, Xingyong Yang, Hong Shen

**Affiliations:** ^1^College of Life Sciences, Chongqing Normal University, Chongqing, China; ^2^Biological Science Research Center, Southwest University, Chongqing, China; ^3^College of Resources and Environment Science, Southwest University, Chongqing, China

**Keywords:** *Bacillus circulans*, upland cotton (*Gossypium hirsutum* L.), disease resistance, growth-promoting, phytohormones synthesis, rhizosphere and endophytic microbiota

## Abstract

Plant growth-promoting bacteria (PGPB) are components of the plant rhizosphere that promote plant growth and/or inhibit pathogen activity. To explore the cotton seedlings response to *Bacillus circulans* GN03 with high efficiency of plant growth promotion and disease resistance, a pot experiment was carried out, in which inoculations levels of GN03 were set at 10^4^ and 10^8^ cfu^⋅^mL^–1^. The results showed that GN03 inoculation remarkably enhanced growth promotion as well as disease resistance of cotton seedlings. GN03 inoculation altered the microbiota in and around the plant roots, led to a significant accumulation of growth-related hormones (indole acetic acid, gibberellic acid, and brassinosteroid) and disease resistance-related hormones (salicylic acid and jasmonic acid) in cotton seedlings, as determined with ELISA, up-regulated the expression of phytohormone synthesis-related genes (*EDS1*, *AOC1*, *BES1*, and *GA20ox*), auxin transporter gene (*Aux1*), and disease-resistance genes (*NPR1* and *PR1*). Comparative genomic analyses was performed between GN03 and four similar species, with regards to phenotype, biochemical characteristics, and gene function. This study provides valuable information for applying the PGPB alternative, GN03, as a plant growth and disease-resistance promoting fertilizer.

## Introduction

In recent years, there has been considerable interest in plant growth-promoting bacteria (PGPB), which are free-living bacteria in the surrounding rhizosphere or in plant. PGPB can improve plant nutrient uptake, enhance plant growth and yield (Lugtenberg and Kamilova, 2009; Hassan et al., 2019), induce resistance against pathogens in plants, and aggressively colonize the root rhizosphere (Lugtenberg and Kamilova, 2009; Emmanuel and Babalola, 2020). The effect of PGPB is primarily explained by their ability to secrete metabolites (Mahmood and Kataoka, 2020) that are able to: (a) stimulate plant growth by inducing the production and release of plant growth regulators or phytohormones such as indole acetic acid (IAA) and gibberellic acid (GA); (b) enhance asymbiotic N_2_-fixation; (c) solubilize inorganic phosphate and mineralize organic phosphate and/or other nutrients; and (d) resist, tolerate, or compete with detrimental microorganisms (Lugtenberg and Kamilova, 2009; Ramakrishna et al., 2019). PGPB also indirectly promote plant growth by improving the diversity of the rhizosphere microbiome and entering inside the roots to trigger induced systemic resistance (ISR), thereby further antagonizing soil borne pathogens (Araujo et al., 2019; Dahmani et al., 2020).

At present, more than 20 species and genera of PGPB strains, including *Agrobacterium*, *Azospirillum*, *Azotobacter*, *Bacillus*, *Burkholderia*, *Enterobacter*, *Klebsiella*, and *Pseudomonas*, have been identified (Aloo et al., 2019). Among them, *Bacillus* is an important genus that can form long-lived, stress tolerant spores existing inside or outside the plant roots under adverse environmental conditions (Gadhave et al., 2018). It has strong saprophytic ability and competitiveness and is capable of secreting metabolites that stimulate plant growth and prevent pathogen infection (Qiao et al., 2017; Aloo et al., 2019; Hashem et al., 2019). Some studies have reported that three principal plant-associated bacteria, *Bacillus cereus*, *Bacillus subtilis* and *Bacillus amyloliquefaciens*, are colonizers of both the in- and outside of plant roots during the growth stage and can improve nutrient utilization, vegetative growth, flowering and fruit ripening, as well as resistance to diseases, insect pests, and environmental stress (Lugtenberg and Kamilova, 2009; Gadhave et al., 2018). However, these studies did not examine the impact of these bacteria on the rhizosphere and endophytic microflora (Qiao et al., 2017); thus, there remains a disconnect between the theoretical research and the practical application of PGPB (Ramakrishna et al., 2019; Alka et al., 2020). For example, the application of a fertilizer with the incorporation of PGPB reportedly showed inconsistent results between laboratory and field applications (Parnell et al., 2016). Currently, limited information is available on the intrinsic relationship between the bacteria and the soil rhizosphere habitat (soil depth, temperature, pH, and intra-species relationships) (Ahemad and Kibret, 2014), further implying that there is a gap in understanding the mechanistic interaction between PGPB and their host plants. In view of this, it is important to determine the impact of these *Bacillus* spp. inoculants on the native microbial community, to effectively apply the theory in field. The potential application of *Bacillus* in the production of a few crops, such as soybean, wheat, and rice (Khatri et al., 2020; Lee et al., 2020; Wang et al., 2020), and its effects on the growth promotion and disease resistance enhancement of upland cotton (*Gossypium hirsutum* L.) are rarely reported.

Upland cotton is an important cultivated oil and fiber crop worldwide; however, it is prone to the attack by the soil-borne hemibiotrophic fungus *Verticillium dahliae* associated with Verticillium wilt (Zhang et al., 2013). As microsclerotia, a dormant survival structure formed by *V*. *dahliae*, can exist in the soil for a long time, Verticillium wilt is considered the most destructive disease of cotton and is extremely difficult to control (Wang et al., 2016; Zhang et al., 2017a). Breeding of upland cotton varieties with disease resistance is limited by more variation in *V. dahliae* strains and the lack of *V. dahliae* resistant germplasm in upland cotton (Zhang et al., 2017a, 2021). Some chemicals, such as benomyl and acibenzolar-*S*-methyl, seem to work for Verticillium wilt, but they are not environment friendly (Goicoechea, 2009). Therefore, application of PGPB to control this disease is a safer and better alternative to protect upland cotton from Verticillium wilt (Hasan et al., 2020). Accordingly, beneficial PGPB with inhibitory action against *V. dahliae* are a promising biocontrol agent for the control of cotton Verticillium wilt. To date, some researchers have isolated multiple strains with biocontrol activities against *V. dahliae* from *Bacillus*, *Enterobacter*, *Paenibacillus*, *Pseudomonas*, *Serratia*, and *Streptomyces* genera (Erdogan and Benlioglu, 2010; Zhang et al., 2018; Cheng et al., 2020; Hasan et al., 2020; Sherzad and Canming, 2020; Tao et al., 2020; Zhang et al., 2020).

*Bacillus circulans* was first isolated and described in 1890 by Jordon. It is a Gram-positive bacterium (Nakamura and Swezey, 1983) with the ability to secrete polysaccharide degrading enzymes such as β-1,3 and β-1,6 glucanases, β-1,3 and β-1,4-glucanases, α-l,3-glucanases, amylase, chitinase, and xylanase (Tanaka and Watanabe, 1995). Several studies have shown that the *B. circulans* strain has the ability to promote plant growth and can be developed as a biological fertilizer (Mehta et al., 2010, 2015; Bokhari et al., 2019).

In this study, upland cotton seedlings were applied as the experimental host plant to investigate the plant growth-promoting mechanism and disease-resistance effect of *B. circulans* GN03 isolated from purple soil. Furthermore, we provided a full overview of the properties attributed by the GN03, and performed its comparative genomic analysis with four other *B. circulans* strains. The results of this study provide useful information for promoting sustainable agricultural practices for improving the soil environment and crop yield while also providing a basis for more in-depth microbial species interaction research.

## Materials and Methods

### Bacterial Strain

The GN03 strain was isolated from the root surface of pakchoi cabbage (*Brassica chinensis*) grown in purple soil in an agricultural field in the Beibei District (30°26′12′′ N, 106°26′25′′ E), Chongqing, China (Shen et al., 2018). Bacterial suspensions were obtained by culturing cells in 50 mL of LB medium (10 g tryptone, 5 g yeast extract, and 10 g NaCl⋅L^–1^) in 300 mL flasks on a rotary shaker (150 rpm) at 37°C for 12 h. Scanning electron microscopy was used for morphological observations (Golding et al., 2016). The spore staining, Gram staining, and biochemical characteristics were evaluated according to Mehnaz et al. (2010).

### Molecular Identification

After culturing GN03 in LB medium for 48 h (37°C, 150 rpm), the bacterial cells were collected by centrifugation (4,500 × *g* for 5 min at 4°C). To preliminarily confirm the strain of bacteria isolated, PCR (94°C for 10 min followed by 34 cycles at 94°C for 30 s, 56°C for 30 s, and 72°C for 90 s, with a final extension at 72°C for 10 min) was performed with universal 16S primers 27F (5′-AGAGTTTGATCCTGGCTCAG-3′) and 1492R (5′-TACGGTTACCTTGTTACGACTT-3′). The PCR products were sequenced by BGI (Shenzhen, China), and the resultant sequences were compared with those hosted on the GenBank database using BLAST. Genomic DNA was isolated from the cell pellets with a Bacteria DNA Kit (OMEGA) according to the manufacturer’s instructions, and quantified using TBS-380 fluorometer (BioSystems, CA).

### Evaluation of Growth Promotion and Disease Resistance in Cotton Seedlings

Bacteria were resuspended and diluted in deionized water (0 cfu^⋅^mL^–1^ for the Mock group, 10^4^ cfu^⋅^mL^–1^ for the C1 group and 10^8^ cfu^⋅^mL^–1^ for the C2 group). Upland cotton (*G. hirsutum* ‘Yumian-1’) seeds kindly provided by Dr. Zhengsheng Zhang (Southwest University, China), underwent surface sterilization with 20% (v/v) H_2_O_2_ for 2 min, and were then individually sown into plastic pots (12 cm diameter and 16 cm height). The soil was collected at 0–15 cm depth from the campus (30°36′45′′N, 106°17′59′′E, 261 m above sea level) of Chongqing Normal University, China. After being sieved (<1 mm) and air-dried, the soil contained 17.19 ± 0.62 g^⋅^kg^–1^ organic matter, 40.76 ± 2.86, 70.19 ± 2.01, and 93.84 ± 10.91 mg^⋅^kg^–1^ available nitrogen, phosphorus, and potassium, 0.78 ± 0.05, 1.02 ± 0.12, and 17.21 ± 0.38g^⋅^kg^–1^ total nitrogen, phosphorus, and potassium, respectively. Once two cotyledons started to unfold, 5 mL GN03 or an equal volume of deionized water was inoculated in rhizosphere (Supplementary Table 1). After 7 days, the plants were re-inoculated using the same method and concentration as in the initial inoculations. Throughout the experimental period, cotton seedlings were randomly placed in greenhouse conditions, wherein the average day/night period, daytime light intensity, temperature, and humidity were 12.5/11.5 h, 2,000–4,000 lux, 16–30°C and 50–80%, respectively. Twenty-three days after the second inoculation, indicators, including phenotypic data, hormone levels, gene expression, and microbial diversity, were measured. Three independent tests with 30 plants per replicate were performed.

The virulent defoliating *V. dahliae* strain V991, stored at −80°C, was first isolated from cotton originated in Xinjiang, China, and reactivated on potato dextrose agar (PDA, 200 g^⋅^L^–1^ potato, 20 g^⋅^L^–1^ dextrose, and 18 g^⋅^L^–1^ agar) medium at 25°C. After growing on PDA medium at 25°C for 7 days, mycelia were collected and cultured in potato dextrose broth (PDB, 200 g^⋅^L^–1^ potato and 20 g^⋅^L^–1^ dextrose) medium for 7 days at 25°C with constant shaking (150 rpm). Once the cotton seedlings presented with two true-leaves, they were inoculated with 5 mL GN03 suspensions (0, 10^4^, and 10^8^ cfu^⋅^mL^–1^, respectively) poured into the soil surrounding the roots. After 24 h, the pre-treated seedlings were inoculated with the V991 strain by injecting 10 mL spore suspensions (10^7^ conidia^⋅^mL^–1^) into the soil around the roots, and control seedlings were inoculated with an equal volume of sterile distilled water (Zhou et al., 2013). The disease index was calculated by assessing 30 individual plants per treatment and repeated three times with the following formula: disease index = Σ (number of disease leaves × disease grade)/(total number of leaves × 4) × 100. The disease grade was classified as follows: 0 (no symptoms), 1 (>0–25% yellowing or wilting leaves), 2 (25–50% yellowing or wilting leaves), 3 (50–75% yellowing or wilting leaves), and 4 (75–100% yellowing or wilting leaves) (Luo et al., 2020). The assessment was performed every 3 days for 21 days post inoculation (dpi) V991.

### Evaluation of Hormone Levels, Growth, and Disease Resistance-Related Gene Expression

The contents of IAA, GA, brassinosteroid (BR), salicylic acid (SA), and jasmonic acid (JA) in cotton leaves were measured using the corresponding ELISA Kits (Shanghai Preferred Biotechnology, China), with minimum detection concentration of less than 0.1 nM and accuracy of more than 99%. To determine the expression of genes related to growth and disease resistance, RNA was extracted from cotton roots using a MiniBEST plant RNA Extraction Kit (TaKaRa, Japan) and a RT reagent Kit for Perfect Real Time (TaKaRa) was used to obtain cDNA. *Ghhistone3* (the gene of *G. hirsutum* histone 3) was used as the reference gene in the quantitative reverse transcription-PCR (RT-qPCR). The expression of growth-promoting and disease resistance-related genes in the cotton root was measured in 10 μL PCR reactions containing 5 μL SYBR Green Real-time PCR Master Mix (Bio-Rad, Hercules, CA, United States), 1 μL root cDNA, 2 μL ultrapure water, and 1 μL each of the 10 μM forward and reverse primers (Supplementary Table 2). The following RT-qPCR protocol was used in a CFX96 instrument (Bio-Rad): 94°C for 2 min followed by 39 cycles of 94°C for 5 s and 60°C for 30 s, then 95°C for 5 s, 65°C for 5 s, and 95°C for 5 s.

### Assessing the Rhizosphere and Endophytic Microbiota

The cotton roots with rhizosphere soils were placed in 0.02 M phosphate-buffered saline (pH 6.8) and incubated at 180 rpm for 20 min. After taking out the plant roots, the suspension was centrifuged at 12,000 × *g* for 10 min to collect the sediment containing the rhizosphere soil samples. The sediment was rinsed with 70% ethanol for 2 min and then with sterile water (5 times), whereas the removed cotton roots from the previous step were analyzed for endophytic microbial content. Total microbial genomic DNA was extracted from both the cotton roots and the rhizosphere soil samples using the DNeasy PowerSoil Kit (Qiagen, Hilden, Germany), following the manufacturer’s instructions, and stored at −20°C for further analysis.

PCR amplification of the bacterial 16S rRNA genes V3–V4 region was performed using the forward primer 38F (5′-ACTCCTACGGGAGGCAGCA-3′) and the reverse primer 806R (5′-GGACTACHVGGGTWTCTAAT-3′) (Xu et al., 2016). PCR amplicons were purified with Agencourt AMPure Beads (Beckman Coulter, Brea, CA, United States) and quantified using the PicoGreen dsDNA Assay Kit (Invitrogen, Carlsbad, CA, United States). After the individual quantification step, amplicons were pooled in equal amounts, and paired-end 2 × 300 bp sequencing was performed using the Illumina MiSeq platform with MiSeq Reagent Kit v3 at Shanghai Personal Biotechnology (China). The data were analyzed on the free online Majorbio Cloud Platform^1^. Operational taxonomic units (OTUs) were clustered at 97% similarity; rarefaction curves (Kemp and Aller, 2004) were prepared using a reasonable amount of sequencing data (Supplementary Figure 1), which produced a flat curve meaning that the amount of sequencing data was large enough to reflect the vast majority of microbial diversity information in the sample. The unclassified bacterial phyla were not applied to analyses. In addition, reads representing chloroplasts were removed prior to further analyses as chloroplasts are abundant in cotton roots.

### Whole Genome Sequencing and Comparative Genome Analysis

The genome was sequenced by Genedenovo (Guangzhou, China) using a combination of the PacBio RS II system (Menlo Park, CA, United States) and Illumina sequencing platforms (San Diego, CA, United States). The GN03 gene model identified by Glimmer V3 was got by the *Ab initio* prediction method (Delcher et al., 2007). Then, all gene models were blasted against the non-redundant (NR)^2^, SwissProt^3^, KEGG^4^, and COG^5^ databases for functional annotation using the BLASTp module. In addition, tRNAs were identified using tRNAscan-SE (v1.23^6^) and rRNAs were determined using RNAmmer (v1.2^7^).

Comparative genomic analysis was carried out by comparing the genome sequence of the GN03 strain with that of four other *B. circulans* stains (Supplementary Table 3). Among them, PK3-109 and PK3-138 strains were isolated from plant root endophytes that grow in the Thar Desert, India (Bokhari et al., 2019); RIT379 strain was isolated from the internal stem tissue of *Costus igneus*, which grows in Puerto Rico (Polter et al., 2015); and a model strain NCTC2610^8^. PK3-109 strain has been shown to produce IAA and exopolysaccharide, and to increase the total fresh weight of *Arabidopsis thaliana* under conditions of salt stress (Bokhari et al., 2019).

### Statistical Analysis

Biochemical and physiological measurements were evaluated using an analysis of variance (ANOVA) followed by Dunnett’s *post hoc* test using the Statistical Package for the Social Sciences, v22.0 (SPSS, Chicago, IL, United States). Means among treatments were considered significantly different when the probability (*p*-value) was less than 0.05. All data in the tables and figures are presented as the mean ± SE (standard error). Values were compared using a one-way ANOVA least significant difference (LSD) test, or a non-parametric Kruskal–Wallis test when the data were not normally distributed. The Bray–Curtis dissimilarity metric and analysis of similarities (ANOSIM) with 999 permutations were performed when comparing groups.

### Data Deposition

16S rDNA amplicon raw sequencing data were deposited in the NCBI Short Read Archive (SRA) BioProject PRJNA631145 under the accession numbers SRR11735611-SRR11735637. The *B. circulans* GN03 genome was deposited under accession numbers CP053315 and CP053316 (the latter is for the plasmid).

## Results

### Morphological, Biochemical and Molecular Characteristics of GN03

**FIGURE 1:**
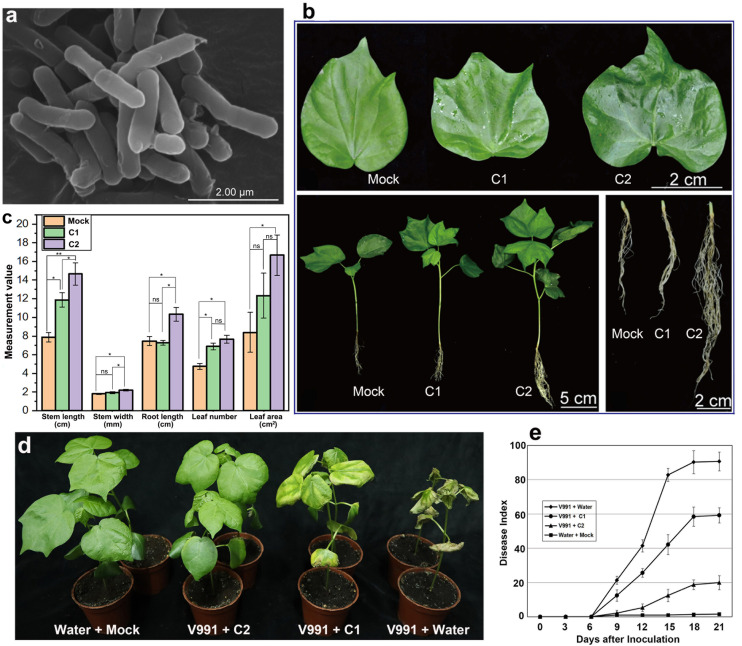
Morphological characteristics and phenotypic changes in cotton seedling 30 days after inoculation of GN03. **(a)** Scanning electron microscopic image of the bacterial cell after culture for 16 h at 37°C. **(b)** The phenotype of the leaf, whole plant and root in cotton seedlings 30 days after inoculation of GN03. **(c)** The biomass of cotton seedlings. Leaf area is measured as the area of the third leaf from the top bud. Statistical significance was evaluated by one-way ANOVA and LSD *post hoc* test (*p < 0.05, **p < 0.01; ns, not significant, *n* = 30). **(d)** Disease symptoms of cotton plants pre-treated with different concentrations of GN03 (0, 10^4^, and 10^8^ cfu/mL) following inoculation with *V. dahliae* strain V991 at 18 dpi. **(e)** Disease indexes of the GN03-pre-treated cotton were determined from 3 to 21 dpi. Values are means ± SD; *n* = 30.

Bacterial characterization of the GN03 strain revealed that it formed white round micro-bulge colonies with a small diameter and neat edges. The cells were rod-shaped and approximately 2 μm long. After fission, the daughter cells separated to form single cells (Figure 1A). GN03 presented as Gram-positive bacterium with oval-shaped mid-spores and capsular hypertrophy (Supplementary Figure 2). Biochemical characterization of GN03 revealed that it produced catalase and nitrate reductase. However, it did not produce phenylalanine deaminase or cytochrome oxidase c, neither degraded tryptophan to produce hydrazine, or use citrate as its sole carbon source. Sugar fermentation tests of GN03 resulted in the production of acid, but not gas. Fermentation of glucose by GN03 resulted in the production of acidic compounds as well as acetyl-methyl-methanol. Furthermore, GN03 was able to hydrolyze starch and liquefy gelatin (Supplementary Table 4). The characteristics of the GN03 strain conformed to the general characteristics of *B. circulans* (Buchanan et al., 1994). Sequencing of 16S rDNA and analysis via NCBI BLAST also revealed GN03 to be a strain of *B. circulans* with the genome characteristics of GN03, similar to the average *B. circulans* genomes recorded by the NCBI. Hence, the complete taxonomic descriptor is: *Firmicutes*, *Bacilli*, *Bacillales*, *Bacillaceae*, *Bacillus*, *B. circulans* GN03.

### Effect of GN03 on Cotton Seedling Growth and Disease Resistance

To explore the plant growth-promoting effect of GN03, each cotton seedling was inoculated with various concentrations of GN03, ranging from 0 cfu^⋅^mL^–1^ as the control (Mock), 10^4^ cfu^⋅^mL^–1^ (C1), to 10^8^ cfu^⋅^mL^–1^ (C2). The evaluated phenotypic characteristics of cotton significantly improved with the increase in inoculation concentration (C2 > C1 > Mock; Figure 1B). Compared to the Mock group, the C2 group exhibited significantly increased stem length, leaf area, root length, stem width, and leaf number by 85.91, 98.45, 38.42, 22.22, and 61.47%, respectively (Figure 1C). Overall, GN03 inoculation promoted cotton seedling growth in a dose-dependent manner.

After pouring different concentrations (0, 10^4^, and 10^8^ cfu^⋅^mL^–1^) of GN03 suspensions into the soil surrounding the roots of the cotton seedling, the seedlings were inoculated with V991 spore suspensions. Different responses to the V991 infection were observed in pre-treated and control seedlings, and the symptoms of wilt and etiolation were much more severe in the control than the pre-treated seedlings at 21 dpi (Mock + V991 > C1 + V991 > C2 + V991; Figure 1D). The disease index increased with prolonged dpi, but disease development was slower in the pre-treated seedlings. At 21 dpi, compared to the Mock + V991 group, the C1 + V991 and C2 + V991 groups exhibited significantly increased Verticillium wilt resistance, and the disease indexes were 90.6, 59.2, and 19.9% for the Mock + V991, C1 + V991, and C2 + V991 groups, respectively (Figure 1E). These results indicated that GN03 inoculation enhanced the disease resistance of cotton seedlings.

### Changes in Phytohormones and the Expression of Disease Resistance-Related Genes After GN03 Inoculation

**FIGURE 2:**
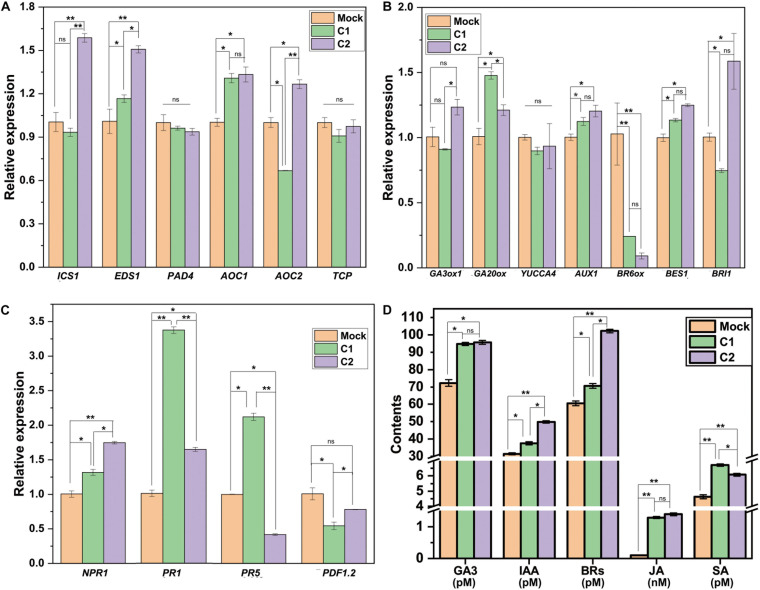
Changes in cotton hormones and related gene expression 30 days after GN03 inoculation. Relative expression of **(A)** SA and JA synthesis-related gene, **(B)** IAA, GA, BR synthesis-related genes, and **(C)** disease resistance-related genes, assessed with RT-qPCR. **(D)** Hormone content of cotton leaves. In the graphs, mean values with SD are indicated; statistical significance was evaluated by one-way ANOVA and LSD *post hoc* test (*p < 0.05, **p < 0.01; ns, not significant, *n* = 5).

*Bacillus* association boosts plant growth and immunity to adversity by regulating growth and stress response genes, proteins, plant hormones, and related metabolites (Radhakrishnan et al., 2017; Afzal et al., 2019; Hashem et al., 2019). Accordingly, in cotton seedling roots, the expression of SA, JA, BRs, and GA synthesis-related genes (*EDS1*, *AOC1*, *BES1*, and *GA20ox*), auxin transporter gene (*AUX1*), and disease resistance-related genes (*NPR1* and *PR1*) was significantly increased (Figures 2A–C). The changes in plant hormone levels in the seedlings were consistent with the expression of genes related to phytohormone synthesis. Compared to those in the Mock group, the level of IAA, GA, BR, JA, and SA were increased by 19.4, 31.1, 16.6, 28.6, and 44.9% in the C1 group, and that were significantly increased by 58.9, 32.4, 68.7, 38.7, and 31.3% in the C2 group, respectively (Figure 2D).

### Rhizosphere Microbiota Changes After GN03 Inoculation

**FIGURE 3:**
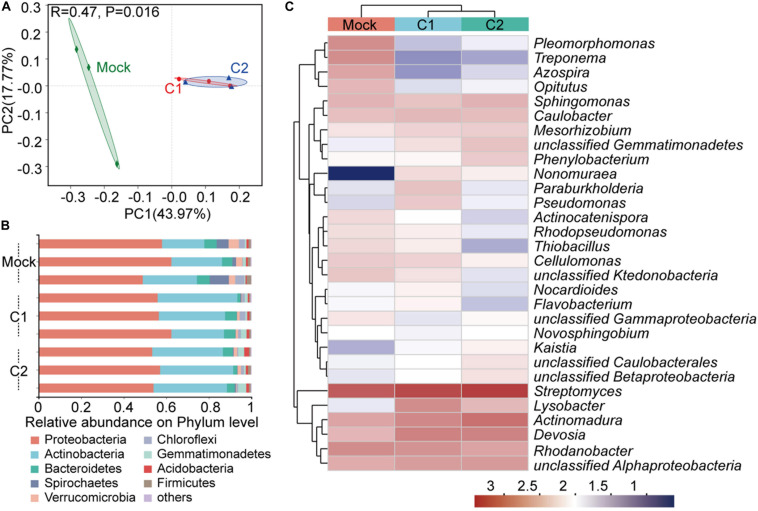
Bacterial community of the rhizosphere 30 days after GN03 inoculation. **(A)** PCoA plot based on the structure of the microbial community. Mock, green diamonds; C1, red circle; and C2, blue triangles. Variation in communities was based on Bray–Curtis distances and an ANOSIM test with 999 permutations. **(B)** Relative abundance of bacterial phyla in different samples. **(C)** Heatmap showing the relative abundance of the dominant taxa with respect to treatment. Hierarchical cluster analysis was based on Bray–Curtis distances using a complete-linkage method. Each sample was measured in triplicate.

Thirty days after inoculation, rhizosphere soil samples from each group were extracted to assess the microbial communities based on 16S rDNA amplicon sequencing. The richness and diversity of bacterial species were evaluated using the Chao1 and Shannon indices (Shannon, 1948; Chao, 1984). The results showed no significantly difference between the rhizosphere sample groups (Supplementary Table 5A). Nevertheless, the PCoA (Ramette, 2007) suggested that the microbiota could be divided into two distinct clusters based on the presence or absence of inoculation (Figure 3A). A network diagram (Faust and Raes, 2012) of species association showed very complex bacterial relationships in the rhizosphere, wherein the bacterial species promoted as well as inhibited each other (Supplementary Figure 3). This indicated that the rhizosphere microbiota was large in number and complex in composition, and that the microbial equilibrium in the rhizosphere would not be easily disrupted, thereby retaining a strong ability to recover.

With regards to rhizosphere bacterial composition at the phylum level (Figure 3B), the top three phyla were Proteobacteria, Actinomycetes, and Bacteroides. The relative proportion of Proteobacteria was approximately 50–60%. In the C2 group, the proportion of Spirochaetes was decreased by 5.05% but that of Actinobacteria was increased by 11.01%. At the genus level, the proportions of *Lysobacter* and *Herbaspirillum*, which have important biocontrol activities and nitrogen fixation abilities (Chubatsu et al., 2012; Exposito et al., 2015), were increased in the rhizosphere after inoculation, while those of *Geobacter*, *Curvibacter*, and *Methylocysti*, which have repair functions (Baani and Liesack, 2008; Gulay et al., 2018), were reduced (Figure 3C and Supplementary Figure 4A).

### Endophytic Microbiota Changes After GN03 Inoculation

**FIGURE 4:**
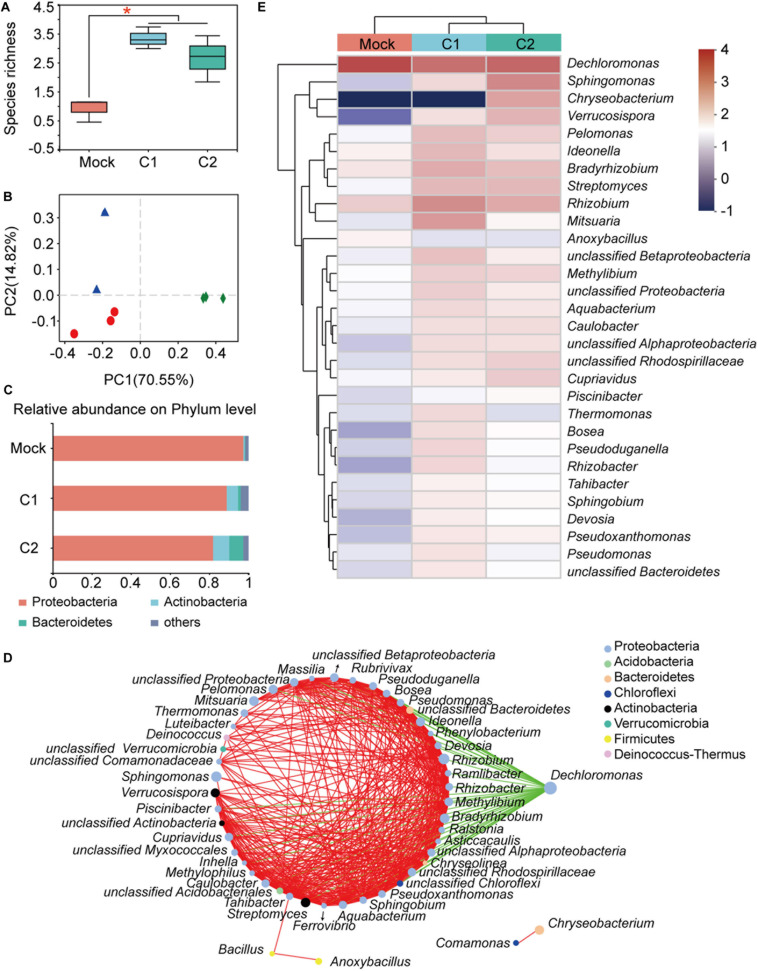
Bacterial community of cotton roots 30 days after GN03 inoculation. **(A)** Boxplot of species richness (number of OTUs) by Shannon index. The asterisk (*) indicates a significant difference (one-way ANOVA, LSD *post hoc* test, *p* < 0.05, see Supplementary Table 5B). **(B)** PCoA plot based on the structure of the microbial community. Each symbol represents a sample. Mock, green diamonds; C1, red circle; and C2, blue triangles. **(C)** Relative abundance of bacterial phyla in each group. Each value is the mean of three measurements. **(D)** Network correlation between plant root bacteria. The nodes on the left side of the ring are positively correlated at all instances (red lines) and the green lines indicate negative correlations between the genera. **(E)** Heatmap showing the relative abundance of the top 30 dominant taxa at the genus level in cotton roots.

The cotton seedlings root samples after GN03 inoculation 30 days from each group were extracted to assess the microbial communities. Corresponding to the rhizosphere results, the microbial diversity in plant roots showed the following changes: (1) species richness and diversity increased after inoculation (Figure 4A and Supplementary Table 5B); (2) each treatment formed a separate cluster in the PCoA (Figure 4B); and (3) the structure of the network diagram was very simple, with only *Dechloromonas* being negatively correlated to the 25 other genera and all others positively correlated to the top 50 genera (Figure 4D). This suggested that the root microbiota was more sensitive to GN03 inoculation than the rhizosphere microbiota. Furthermore, functional prediction also confirmed this result. PICRUSt2 software (Douglas et al., 2019) analysis showed that the clusters of orthologous groups (COG) of protein function did not change significantly in the rhizosphere. However, in post-inoculation plant roots, amino acid transport and metabolism were the most abundant functions, followed by energy production. Furthermore, fatty acid metabolism, the pentose phosphate pathway, and biosynthesis of neomycin, kanamycin, and gentamicin increased following GN03 inoculation, while plant apoptosis and plant disease were inhibited. Further testing was performed to identify the bacteria responsible for this functional change.

The top bacterial species at the phylum level of roots, were similar to those in the rhizosphere (Figure 4C). However, their relative abundances varied greatly. For example, the relative proportion of Proteobacteria reached 97.46% within roots, whereas it was 52.63% in the rhizosphere. It should be noted that, as the inoculation concentration increased, the relative proportion of Proteobacteria gradually decreased to 81.94%. In the C2 group, the proportions of Actinobacteria and Bacteroidetes were increased by 7.65 and 6.87%, respectively. At genus level, the proportion of many genera increased after GN03 inoculation, especially in the C1 samples (Figure 4E and Supplementary Figure 4B), including *Rhizobium*, a bacterium that forms a symbiotic relationship with plant roots and is able to fix nitrogen (Zilli et al., 2020), *Rubrivivax*, a photosynthetic bacterium (Mekala et al., 2019), *Actinomadura*, a bacterium with potential use in new antibiotics (Lazzarini et al., 2000), and *Labrys*, a bacterium capable of chlorobenzene and fluoride biodegradation (Amorim et al., 2014). These results indicated that GN03 regulated the changes in the rhizosphere and endophytic microbiota. After inoculation, the microbial community had a huge change and showed some regularities.

### Comparative Genomic Analysis of GN03

**FIGURE 5:**
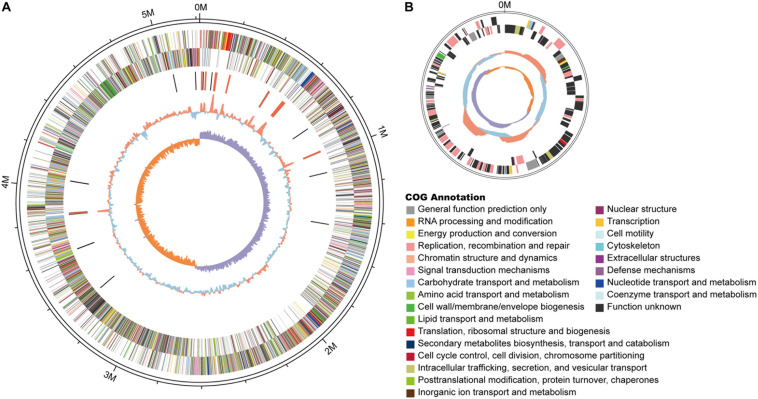
Schematic of the complete GN03 genome. GN03 chromosome **(A)** and plasmid **(B)**. Rings represent the following features labeled from the outside to the center, where the outermost circle represents the scale in bp: the first ring represents positive strand genes and the second ring represents negative strand genes. Each color patch represents a COG functional classification. The third ring represents ncRNA (black indicates tRNA, red indicates rRNA). The fourth ring represents the GC content (red indicates GC content above the mean, blue indicates GC content below the mean). The innermost ring shows the GC skew (GC skew = [GC]/[G + C]; purple means greater than 0, orange means less than 0). Circos v0.62 (http://circos.ca/) software was used to draw the genomic circle map.

The above findings have so far demonstrated that GN03 inoculation can promote cotton seedlings growth, change the microbiota in and around the plant roots, increase phytohormones in cotton seedlings, up-regulate the expression of phytohormone synthesis and disease-resistance related genes. Then, we conducted a genome-wide sequencing analysis followed by a comparative genome analysis. The complete genome sequence of GN03 was deposited in the GenBank database (chromosome and plasmid accession numbers CP053315 and CP053316, respectively). An illustration of the genomic structure of the GN03 chromosome is shown in Figure 5A, and that of the plasmid is shown in Figure 5B. The total genome length was 5,217,129 bp. The mean average GC content was 35.64%, which was similar to the average GC content across *B. circulans* genomes (length, 5.09 Mbp; mean GC content, 35.5%) recorded by the NCBI. Of interest, GN03 contained a plasmid that was 0.18 Mbp long with a mean GC content of 31.62%. Multiple databases (Nr, COG, KEGG, Swiss-Prot, GO, and CAZy) were integrated to annotate gene function through sequence alignment.

The GN03 genome included clusters of the following: *de novo* amino acid synthesis and metabolic pathways, *de novo* sugar synthesis and metabolism (such as starch, sucrose, and other polysaccharide metabolism), and *de novo* lipid synthesis (such as saturated fatty acids and unsaturated fatty acids synthesis) and metabolism. It also contained gene clusters for folic acid synthesis and for porphyrin, chlorophyll, butyric acid, biotin, inositol phosphate, sulfur, and methane metabolism. GN03 could effectively degrade naphthalene, xylene, ethylbenzene, nitrotoluene, chlorobenzene, and chlorocyclohexane. The genome also contained a comprehensive DNA repair system that could act as a DNA protectant. The functions of plasmid genes were mainly focused on DNA replication, mismatch repair, and phosphonate metabolism. In addition, we predicted the presence of genes that confer resistance by producing antibiotics (streptomycin, surfactin, penicillin, novobiocin, and cephalosporin) and other genes, such as *gabD, opuC* and *opuA* and *proX, proV*, and *proW*, against stress. These results indicated that GN03 was capable of pathogen resistance and pollutant degradation.

**FIGURE 6:**
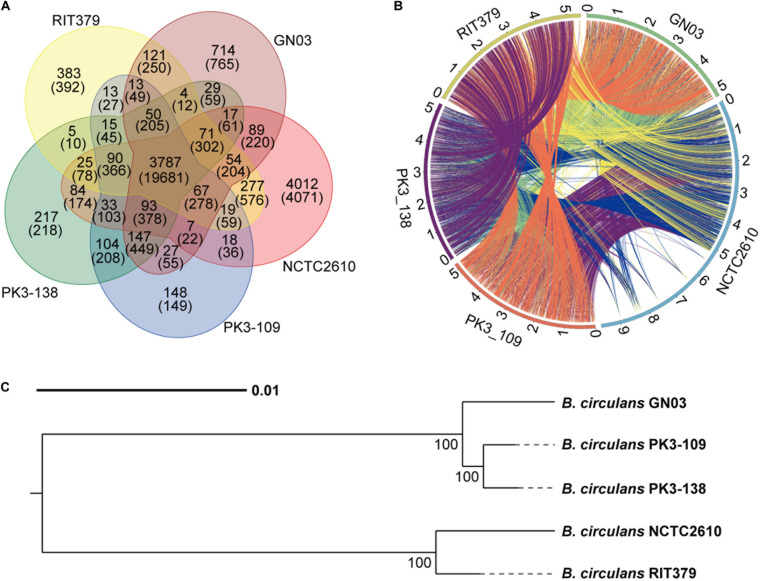
Comparative genomic analysis of GN03 with other four *Bacillus* stains. **(A)** Venn diagram showing the number of genes in all five strains. In BLAST alignment, gene pairs with *E*-values less than 1e-7 are considered to be homologous. OrthoMCL was used to group the homologous genes into the same families. The upper number represents the number of gene families and the lower number represents the number of genes. **(B)** The collinear circos diagram of the five bacterial strains. The outermost circle represents the genomic sequences of the five bacteria. For example, green in the upper right corner represents the genomic sequence of GN03 bacteria. Every two strains are connected by a line representing a collinear area between the two bacteria. **(C)** Phylogenetic tree of the five strains. The tree was built using a single copy homologous gene using the neighbor joining method with a bootstrap value of 1,000. Unit: nucleotide substitution rate.

We selected four *B. circulans* reference strains with or without growth-promoting function (Supplementary Table 3) and performed a comparative genomic analysis. To identify homologous genes and specific gene families among the five bacterial species, homologous genes were identified using multiple databases (with an *E*-value < 1e-7) and the numbers of genes and gene families were analyzed using a Venn diagram (Figure 6A). Functional annotation data were obtained from multiple databases. The following top three KEGG pathways were shared among all strains: secondary metabolite biosynthesis, ribosome biosynthesis, and aminoacyl-tRNA biosynthesis. The collinear and the molecular evolutionary tree analysis of GN03 with four reference strains (Figures 6B,C) showed that the GN03 strain is most closely matched to the PK3-109 strain, which has also been reported to promote plant growth (Bokhari et al., 2019).

## Discussion

### GN03 Inoculation Promoted Cotton Seedling Growth and Accumulation of Growth- Related Phytohormones

Plant roots can anchor and absorb nutrients and water in the soil. Lateral roots and root hairs are closely related to nutrient and water use efficiency. Many PGPB strains are known to promote the increase of lateral roots and the formation of root hairs (Zamioudis et al., 2013; Luo et al., 2019). GN03 is a soil-borne bacterium with positive effects on plant growth (Shen et al., 2018). In this study, The GN03 promoted the growth of cotton seedlings, which may be achieved by increasing the length and number of lateral roots (Figures 1B,C) resulting in cotton seedlings with a larger root surface in contact with the soil, thus boosting plants to absorb more nutrients and water. Accordingly, the GN03-treated cotton seedlings showed obvious increases stem length and width, leaf number and area during growth, especially in the C2 group (Figures 1B,C).

The improving effect of PGPB on plant growth is mainly reflected in the production of hormones or the induction of hormone signals (Egamberdieva et al., 2017). Phytohormones, such as IAA, GA, and BRs, can regulate various cellular processes and plant responses to biotic and abiotic stresses with a very low concentration (Ciura and Kruk, 2018). Auxins play an essential role in plant developmental process ranging from the cell division, and cell elongation to morphogenesis (Felten et al., 2009). Brassinosteroids are capable of executing diverse functions in plant growth, development, and stress tolerance (Banerjee and Roychoudhury, 2018). Gibberellins play an indispensable role in stem elongation, leaf expansion, and plant development, while GA deficit results in plant dwarfism (Ciura and Kruk, 2018). In our study, GN03 inoculation led to accumulation of growth-related hormones (IAA, GA, and BRs), as determined with ELISA, in the cotton leaves. Compared to those in the Mock group, the level of IAA, GA, and BR were increased by 19.4, 31.1, and 16.6% in the C1 group, and that were significantly increased by 58.9, 32.4, and 68.7% in the C2 group, respectively (Figure 2D). Accordingly, compared to those in the Mock group, the expression of IAA, BRs, and GA synthesis-related genes (*AUX1*, *BES1*, and *GA20ox*) was significantly increased in cotton seedling roots (Figure 2B). Phytohormones can be transported to different parts of the plant by different sophisticated transporter molecules through the vascular system in plant (Lacombe and Achard, 2016). These hormones may include endogenous hormones produced by the plant and the exogenous hormones produced by rhizosphere and endophytic microbes (Backer et al., 2018). Hence, this may also be the reason for the total amount of hormones accumulated in the leaves.

### GN03 Inoculation Promoted Cotton Seedling Disease Resistance and the Expressing of Disease Resistance-Related Genes

The plant hormones JA and SA play key roles in the response of the plant to *V. dahliae* infection (Glazebrook, 2005; Zhang et al., 2017b). Consistently, we observed significant differences in JA and SA levels (Figure 2D) and changes in the expression of both JA and SA synthetic genes (*EDS1* and *AOC1*, respectively) and disease resistance-related genes (*NPR1* and *PR1*) after inoculation with the GN03 strain. Meanwhile, disease assessment after GN03 pre-treatment revealed significantly increased cotton disease resistance to *V. dahliae* (Figures 1D,E). We, therefore, presume that this resistance is mediated by GN03-regulating hormones signaling pathways. However, further molecular and genetic evidence is needed to elucidate the resistance mechanisms. Controlling Verticillium wilt is challenging, since the pathogen *V. dahliae* can survive for many years in the soil in dormant survival structure (microsclerotia) forms, which germinate to produce hyphae approximal to the root exudates of a suitable host (Inderbitzin et al., 2011; Inderbitzin and Subbarao, 2014; Luo et al., 2014; Daayf, 2015; Shaban et al., 2018). Some bacteria, collectively known as PGPB, can be effective when applied as fertilizers. This is of great significance for the development of more environment friendly agricultural practices. Moreover, biological control agents are eco-friendly strategies with great promise for controlling Verticillium wilt in cotton (Markakis et al., 2016; Zhang et al., 2021). *B. circulans* GN03 can promote upland cotton seedling growth and significantly reduce the incidence of Verticillium wilt. To our knowledge, this study is the first to investigate the growth promotion of *B. circulans* in cotton and application of *B. circulans* cells to reduce disease incidence.

Several beneficial bacteria and non-pathogenic rhizobacteria can also cause activation of the SA/JA pathway and lead to hormonal changes (Olanrewaju et al., 2017). After inoculation, the abundance of bacteria showed additional beneficial function in the roots, especially in the C1 group, though the C2 group showed a stronger growth promotion effect than C1. The expression levels of hormone-related genes (endogenous hormones) in plant roots support the theory that plants can reduce their growth to invest in disease resistance. This implies that the activation of plant growth and defense against potential pathogens are opposing processes in that the onset of one often leads to the inhibition of the other (Zhu et al., 2013; Huot et al., 2014).

To infect cotton roots favorably, *V. dahliae* can selectively steer the local microbiome by its effector proteins, and increasing reports have shown that the microbiome inside and outside a plant plays an crucial role in its health (Snelders et al., 2020). *Bacillus* species can produce some antibiotics, which can directly strangle the growth of the pathogen or induce systemic resistance in plants (Hasan et al., 2020). In our study, it was found that GN03 possess antibiotics (streptomycin, surfactin, penicillin, novobiocin, and cephalosporin) encoding genes and other genes, such as *gabD, opuC* and *opuA* and *proX, proV*, and *proW*, against stress. In addition, GN03 inoculation altered the microbiota in and around the plant roots and the expressing of disease resistance-related genes. Finally, the co-production of these antibiotics producing genes and the changes in cotton seedlings after GN03 inoculation may be form a amassing role against *V. dahliae*.

### GN03 Inoculation Changed the Rhizosphere and Endophytic Bacterial Composition

Within the complex soil environment, many biotic and abiotic factors affect the colonization of PGPB. As colonization can be enhanced by multiple inoculations (Benizri et al., 2001; Coy et al., 2014), we tested different inoculation concentrations (C1 and C2), with greater inoculation concentration showing better beneficial effects. Furthermore, it was previously shown that rhizosphere microorganisms can recover after being temporarily affected by PGPB inoculation (Qiao et al., 2017). Our study confirmed that there were no significant differences in rhizosphere, while various plant indicators showed significant differences. We speculate that GN03 exerted a high ecological effect, which significantly influenced other bacterial changes within 30 days, such as increase in the levels of *Lysobacter, Rubrivivax*, and *Rhizobium* that are beneficial in promoting plant growth and enhancing disease resistance. Interestingly, obvious change of *Bacillus* or Firmicutes in the cotton seedling roots was found, while that was not found around the cotton seedling roots (Supplementary Figure 5). This may be due to (1) the time interval of detection is too long (30 days after inoculation); (2) microbial competition in a wide diversity of niches (Snelders et al., 2020). The dynamic changes of bacterial composition (especially beneficial bacteria including *Bacillus*) discovered in the 30-day period after inoculation need to be further investigated in future research.

Activation of defense against potential pathogens may inhibit growth activation, whereas the abundance of beneficial bacteria results in either the generation or stimulated-release of growth hormones (Backer et al., 2018). In general, plants in the C1 group did not show excessive growth promotion or inhibition which indicates that GN03 concentration is crucial to achieve optimal growth promotion effects.

### Genome Analysis of GN03 Promoting Plant Growth and Disease Resistance

Our study demonstrated that GN03 promoted plant growth by regulating the rhizosphere and endophytic microbiota, together with the expression of plant hormones. To further understand the underlying mechanism, we conducted a genome-wide sequencing analysis, followed by a comparative genome analysis, to elucidate this growth promoting effect. Various genes of interest were found to be involved in the potential growth promoting and disease-resistance effects observed with GN03 inoculation. Inorganic phosphate uptake in some beneficial bacteria may be promoted by high-affinity phosphate transport systems regulated altogether by *PstB, PstC, PstA*, and *PstS; PhnC, PhnE*, and *PhnD* (Liu et al., 2016), and the genes *cysC, cysI, cysJ, cysH*, and *cysN*, responsible for H_2_S biosynthesis (Dooley et al., 2013). Of note, some of the *trp* cluster genes (*trpA, trpB, trpD*, and *trpC*) involved in tryptophan biosynthesis might be involved in multiple biological processes, including plant hormone biosynthesis (Gupta et al., 2014). The *speB* and *speE* genes encode agmatinase and spermidine synthase to catalyze the transformation of amino acids into plant growth-promoting compounds (Cassan et al., 2009). Furthermore, *gabD* is responsible for the production of pest/disease inhibiting γ-aminobutyric acid (Gupta et al., 2014). Autoinducer-2 (*luxS*) is a small molecule, produced by a number of bacterial species, that is implicated in the regulation of biofilm formation, motility, and the production of virulence factors. It has been reported to act directly through quorum sensing or indirectly through modulation of cellular metabolism (Reading and Sperandio, 2006). Lastly, the heat-shock protein genes *dnaK* and *groEL* could aid the survival of GN03 in harsh environments (Guazzaroni et al., 2013), whereas *opuC* and *opuA* and *proX, proV*, and *proW* might be involved in protecting plants against oxidative stress (Gupta et al., 2014).

In conclusion, the GN03 strain of *B. circulans* has been shown to improve plant growth and disease resistance, but the exact underlying mechanism is unclear. In fact, to our knowledge, very little work has been done on the synergistic relationship of plant root microbiota and *B. circulans.* This study showed that the application of *B. circulans* GN03 in cotton seedlings can regulate the microbiota in and around the plant roots, promote cotton seedling growth and disease resistance, induce the production of phytohormones such as IAA, GA, and SA, and up-regulate the expression of phytohormone synthesis-related genes (*EDS1*, *AOC1*, *AUX1*, *BES1*, and *GA20ox*) and disease-resistance genes (*NPR1* and *PR1*).

## Data Availability Statement

16S rDNA amplicon raw sequencing data were deposited in the NCBI Short Read Archive (SRA) BioProject PRJNA631145 under the accession numbers SRR11735611–SRR11735637. The *B. circulans* GN03 genome was deposited under accession numbers CP053315 and CP053316 (the latter is for the plasmid).

## Author Contributions

LQ designed and performed the experiments. XY conducted the experiments. QC and WJ performed cotton growth measurements. SH, PT, and CX performed RNA extraction, RT-qPCR, and assisted in bioinformatics data analysis. LQ, PT, and QC analyzed the data, wrote the original draft of the manuscript, and performed the lab work. LQ, PT, and XY reviewed and edited the manuscript. XY obtained the funding. All authors read and approved the final manuscript.

## Conflict of Interest

The authors declare that the research was conducted in the absence of any commercial or financial relationships that could be construed as a potential conflict of interest.

## Footnotes

1 http://www.majorbio.com

2 https://ftp.ncbi.nih.gov/blast/db/

3 http://uniprot.org

4 http://www.genome.jp/kegg/

5 http://www.ncbi.nlm.nih.gov/COG

6 http://lowelab.ucsc.edu/tRNAscan-SE

7 http://www.cbs.dtu.dk/services/RNAmmer

8 https://www.ncbi.nlm.nih.gov/biosample/?term=circulans%20NCTC2610
