# Supplementary material for: Bacillus circulans GN03 Alters the Microbiota, Promotes Cotton Seedling Growth and Disease Resistance, and Increases the Expression of Phytohormone Synthesis and Disease Resistance-Related Genes
Source: Front Plant Sci. 2021 Apr 14;12:644597. doi: 10.3389/fpls.2021.644597 (PMC8079787; doi:10.3389/fpls.2021.644597)
Supplement: Supplementary file 1 [file Data_Sheet_1.pdf]

Bacillus circulans GN03 alters the microbiota to promote cotton seedling growth and disease resistance via the expression of phytohormone synthesis and disease resistance-related genes

Lijun Qin[1,2,#], Peidong Tian[1#], Qunyao Cui[1], Shuping Hu[1], Chengjian Xie[1], Wei Jian[1], Xingyong Yang[1*], Hong Shen[2,3*]

1. College of Life Science, Chongqing Normal University, 401331, China
2. Biological Science Research Center of Southwest University, 400715, China
3. College of Resources and Environment, Southwest University, 400715, China

Running Head: Plant growth and *Bacillus circulans* GN03

[#]These authors contributed equally to this work.

*Address correspondence to Xingyong Yang（yangxy94@swu.edu.cn) and Hong Shen (shenhong@swu.edu.cn).

Supplementary Material

Supplementary Figures

Figure S1: Rarefaction curves of microbiota. (**a**) the bacterial at rhizosphere, (**b**) in cotton roots. Plot with Shannon index at OTU level of 97% similarity

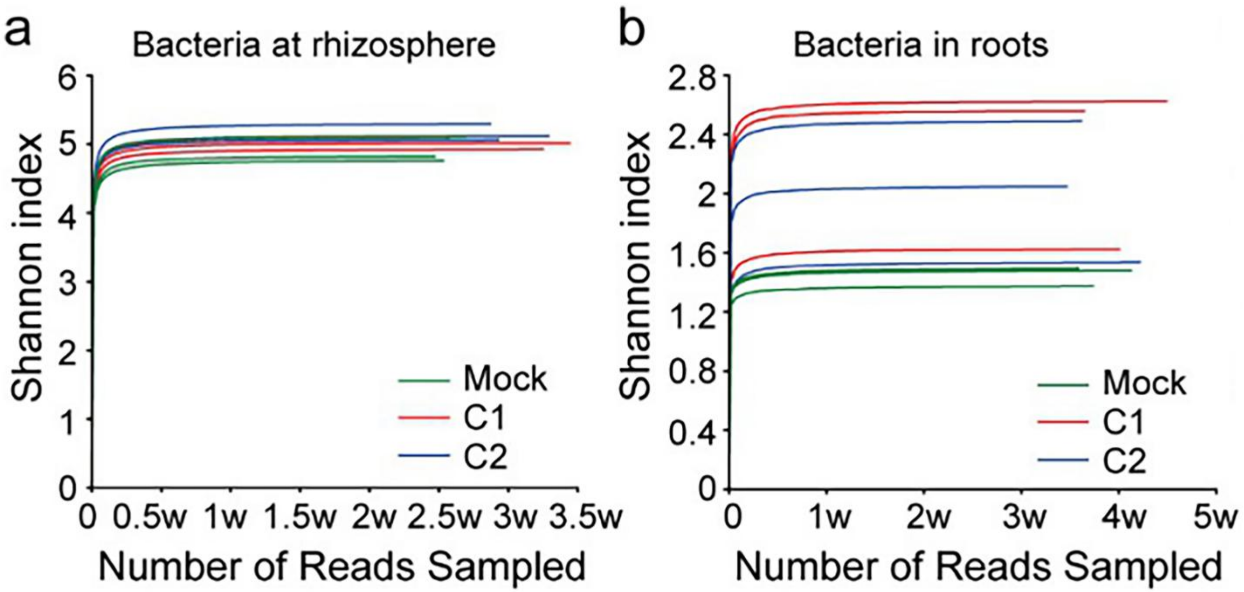

Figure S2: Morphological characteristics of *B. circulans* GN03. (**a**) Gram stain of GN03. Blue-purple is positive and red is negative. (**b)** Spore stain of GN03. The spores are green and the cells are red. Cultivated for 16 h at 37 °C. bar = 10 μm.

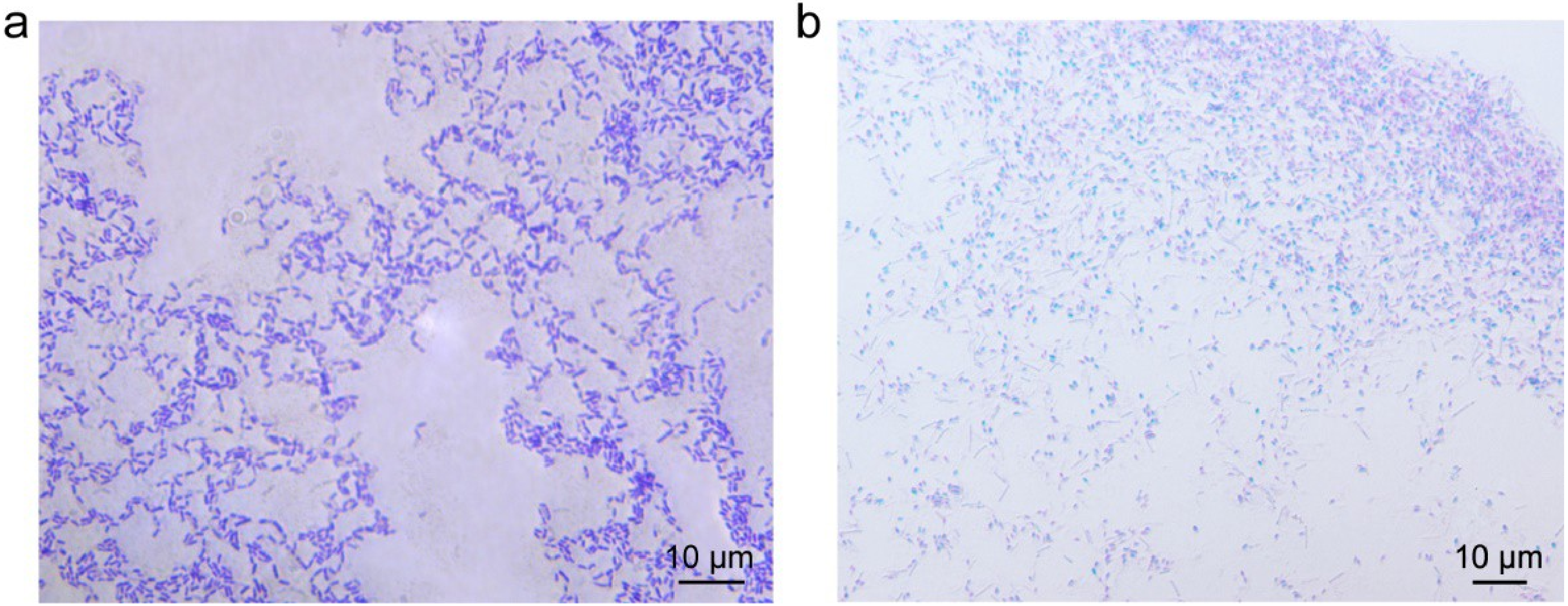

Figure S3: Network correlation of rhizosphere bacteria. Select the top 50 species with a total taxonomic level of abundance, and calculate correlation coefficients Pearson rank between species to reflect the correlation between species. the species with p ≤0.05 are shown, the size of the nodes indicates the species abundance, and different colors indicate different Phylum. Red line indicates positive correlation, and green indicates negative correlation; the thickness of the line indicates the size of the correlation coefficient, and the thicker the line, the higher the correlation between species. The more lines the species have, the closer other species.

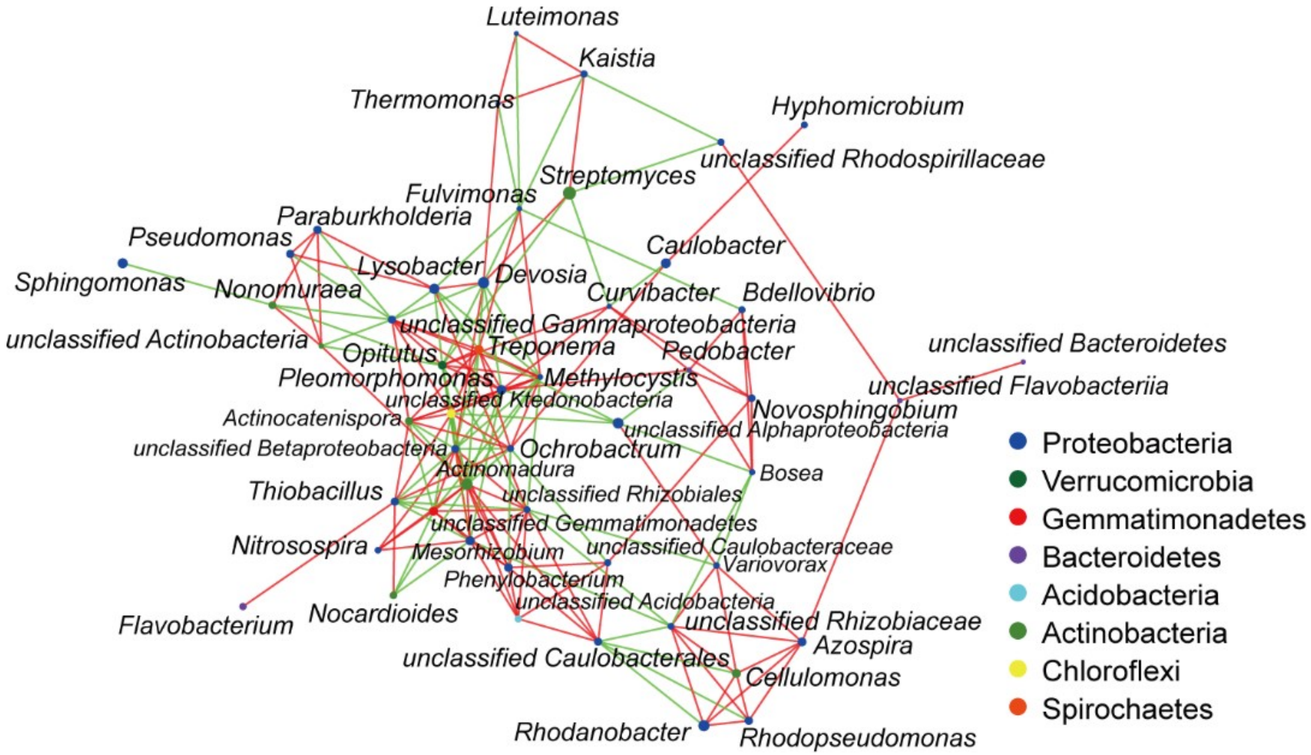

Figure S4: Species difference analysis based on LEfSe (http://huttenhower.sph.harvard.edu/galaxy/root?tool_id=lefse_upload) at genus level. (**a**) the bacterial at rhizosphere, (**b**) in cotton roots. Linear discriminant analysis (LDA) was used to estimate the effect of the abundance of each component (species) on the difference effect. LDA threshold was 2, and Multi-group comparison strategy was all-against-all (more strict) that only species with differences in multiple groups can be considered as different species.

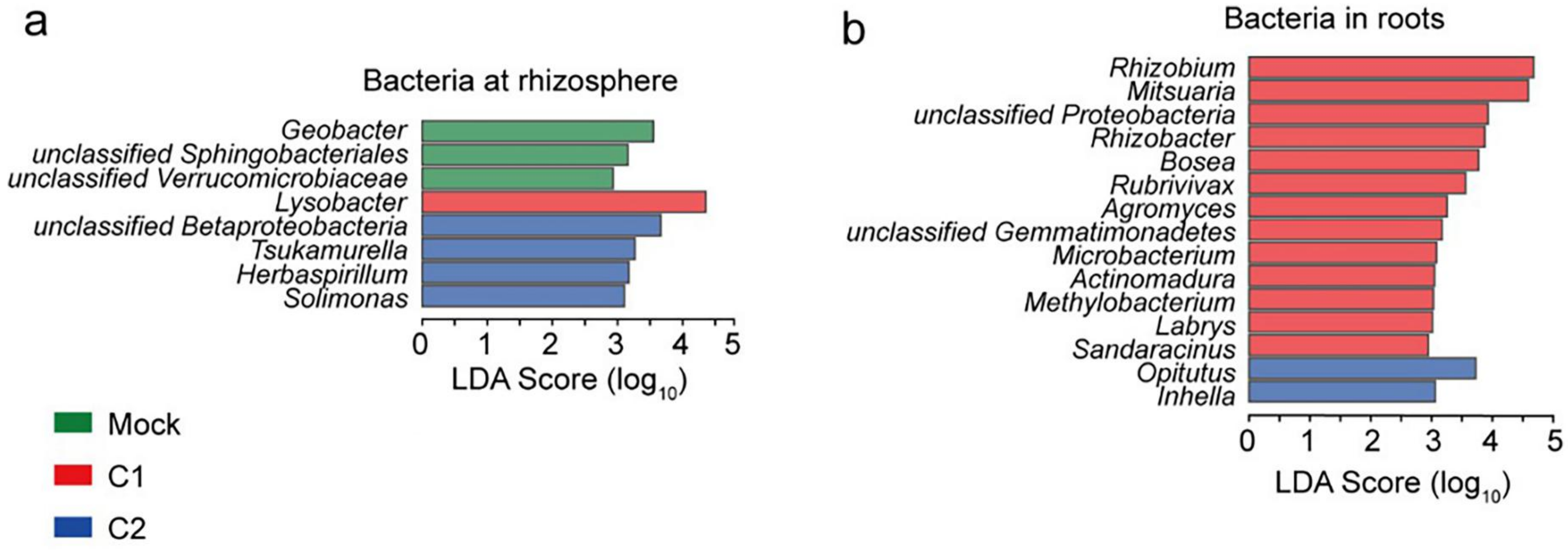

Figure S5: The changes of *Bacillus* species and Firmicutes in and around the cotton seedling roots. (**A**) *Bacillus* species at rhizosphere, (**B**) *Bacillus* species in cotton roots, (**C**) Firmicutes at rhizosphere, (**D**) Firmicutes in cotton roots.

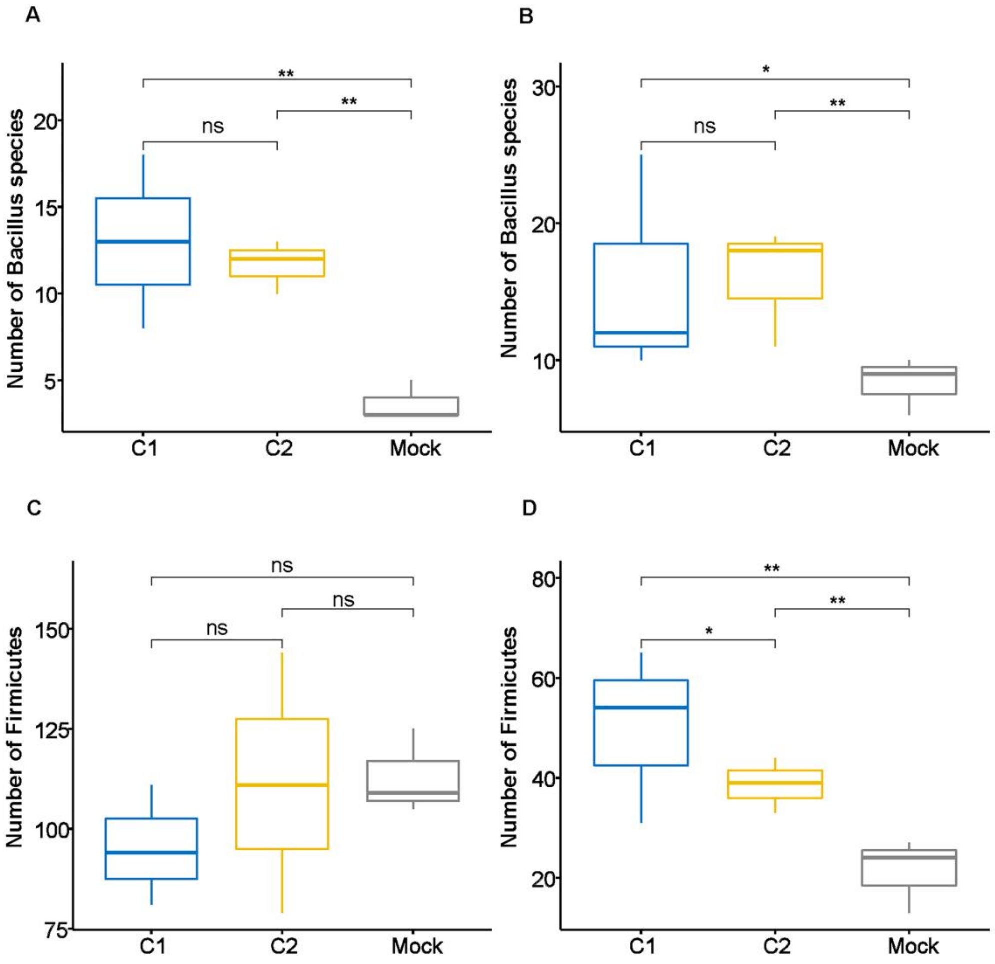

Supplementary Tables

Table S1: Experimental design of cotton growth-promoting effected by inoculation GN03.

Group	Method（1 mL/per plant）
Mock	Sterile water
C1	Inoculation 10^4 bacterial cells/mL GN03 twice
C2	Inoculation 10^8 bacterial cells/mL GN03 twice

Table S2: Primers for RT-qPCR

Gene name	Forward primer (5'--3')	Reverse primer (5'-3')	reference
Histone3	GCCAAGCGTGTCACAATTATGC	ACATCACATTGAACCTACCACTACC	Tu et al., 2007
AUX1	TTAACCCTTCCATCCGCTTC	TCTCCCACACGAAGTACAATG	Yang et al., 2006
GA20ox	TTTACAAGAGCCGCTTGCAC	ACAAAGCCCAGCATTGTCC	Yang et al., 2006
BR6ox1	GACAGGACCAAAGAGATGGCT	TCTTGCCTGAAATCCTCGACG	Yang et al., 2006
BES1	GGCTTGAGGCAATACGGTAATT	AGGCGGCTAGCACATCGTT	Yang et al., 2006
BRI1	CTTAAGACGCTTGATCTCAGCTACA	CGGCAAGGTTCCGATTGA	Li et al., 2013
ICS1	ATGGATGAATGGGTGCGAAGG	AAGAATGCCAGAGGTAAGAGGAGGA	Sun et al., 2014
EDS1	GCAGCAACAGCTCCTCTACCTCAA	GGCAGACCAAGACGCTACAGATACA	Sun et al., 2014
AOC1	CTCCCAAGCCCACAAAAGTT	ACAGAGTCCAGCCGTGATGC	Sun et al., 2014
AOC2	ATCTACTTCCACTCTTCCT	GCCATTCCTTCTTCTTCTA	Wang et al., 2015
TCP	CATCCGAACCCCAGATATGG	TGTAAGGGTGCTTGCAATGTG	Wang et al., 2015
NPR1	CTAGCTTGCGGAGGGATTGATACC	GAGATGGCTGACCTGTCAAACTGC	Yan et al., 2016
PR1	AAGAATGTGGGTTAGTGAGAGGGT	ACCACTTGAGTATAATGCCCGC	Yan et al., 2016
PR5	AAGGAGTCCACCACAATCACCG	CTGGCACTGCTATGGCTCGTAT	Li et al., 2018
PDF1.2	CTGTGGTAGCGGATGGTGATAAG	GTGCAGACGCATTTGCGAAGGAA	Li et al., 2018

References:

Li, X., et al., A cotton cyclin-dependent kinase e confers resistance to *Verticillium dahliae* mediated by jasmonate-responsive pathway. Front Plant Sci, 2018. **9**: p. 642.

Li, Y., et al., *A* cotton gene encoding MYB-like transcription factor is specifically expressed in pollen and is involved in regulation of late anther/pollen development. Plant Cell Physiol, 2013. **54**(6): p. 893-906.

Sun, L., et al., Cotton cytochrome P450 CYP82D regulates systemic cell death by modulating the octadecanoid pathway. Nature Communications, 2014. **5**(1): p. 5372.

Tu, L., et al., Suitable internal control genes for qRT-PCR normalization in cotton fiber development and somatic embryogenesis. Chin Sci Bull, 2007. **52**(22): p. 3110-3117.

Wang, L., et al., Comparative transcriptomics reveals jasmonic acid-associated metabolism related to cotton fiber initiation. PloS one, 2015. **10**(6): p. e0129854.

Yan, Z., et al., Island cotton enhanced disease susceptibility 1 gene encoding a lipase-like protein plays a crucial role in response to *Verticillium dahliae* by regulating the SA level and H_2O_2 accumulation. Front Plant Sci, 2016. **7**: p. 1830.

Yang, S., et al., Accumulation of genome-specific transcripts, transcription factors and phytohormonal regulators during early stages of fiber cell development in allotetraploid cotton. Plant J, 2006. **47**(5): p. 761-75.

Table S3: General information of *B. circulans* GN03 and other four *B. circulans* strains. Parentheses indicated the number of comments in plasmid.

B. circulans Strain	Genome size	GC content	tRNA	5S rRNA	16S rRNA	23S rRNA	plasmid	Transponson	GI	Accession no.	characteristic
GN03	5398834	35.50%	84	11	11	11	1	25 (3)	23 (1)	CP053315<br>CP053316	Rhizobacteria; promote plant growth
PK3-138	5274417	35.67%	82	11	11	11	0	2	9	CP026033	Plant root endophytes; produce IAA
PK3-109	5175203	35.56%	82	11	11	11	0	2	11	CP026031	Plant root endophytes; increase fresh weigh of plant
RIT379	5454271	35.37%	85	10	1	11	0	14	17	LDPH0000000	internal stem tissue of the insulin plant
NCTC2610	9629443	39.83%	154	17	18	18	0	20	21	UAPZ0000000	

Table S4: Physiological and biochemical characteristics of GN03. All test strains were cultivated for 16 h at 37 ℃. Note： “＋”present positive reaction，“－”present negative reaction.

Strain	GN03
Catalase test	＋
Oxidase test	－
Starch hydrolysis test	＋
D-glucose produced acid test	＋
D-xylose produced acid test	＋
D-glucose produced gas test	－
methyl red test	＋
Voges-Proskauer test	＋
Citrate utilization test	－
Indole test	－
gelatin liquefaction test	＋
phenylalanine deaminase test	－
nitrate reductase test	＋

Table S5: Alpha diversity for Shannon, Chao, Sob, and etc index for different test group and variance analysis between groups by Student's test in rhizosphere bacteria (**a**), cotton roots bacteria (**b**).

Symbol	Estimators	Mock-Mean	Mock-Sd	C1-Mean	C1-Sd	C2-Mean	C2-Sd	Pvalue (C1-C2)	Pvalue (Mock-C1)	Pvalue (Mock-C2)
	sobs	227.33	10.263	237.33	3.0551	241.67	14.572	0.6407	0.1811	0.2361
(a)	shannon	4.1052	0.099157	4.1682	0.089615	4.1669	0.08276	0.9857	0.46	0.4548
	ace	252	18.968	263.76	6.3512	268.34	15.013	0.6517	0.3659	0.3068
	chao	250.15	20.266	265.46	11.143	271.75	5.9265	0.4362	0.3156	0.1511
	coverage	0.99578	0.00088695	0.99566	0.0004079	0.99555	0.00029246	0.7094	0.8466	0.6888
	sobs	90	16.462	155	11.79	135.67	21.502	0.2438	0.005123	0.04321
(b)	shannon	0.90966	0.39989	3.3392	0.37629	2.6654	0.79995	0.2573	0.001558	0.02727
	ace	152.32	18.438	201.94	4.0165	167.25	30.964	0.1266	0.01038	0.5128
	chao	121.09	11.228	199.67	4.3078	162.49	36.694	0.1562	0.0003473	0.135
	coverage	0.99092	0.0004119	0.98795	0.0007783	0.99065	0.0020601	0.1012	0.004296	0.8348
	sobs	2.3333	0.57735	3.6667	1.1547	3.3333	1.5275	0.778	0.1481	0.3486
